# Anxiety among the General Population of Himalayan District during the COVID-19 Pandemic: A Descriptive Cross-sectional Study

**DOI:** 10.31729/jnma.6553

**Published:** 2022-02-28

**Authors:** Sanjib Pandit, Niresh Thapa, Asha Sharma, Bishal Pokhrel, Gajendra Bham, Nandaraj Acharya

**Affiliations:** 1 Rapti Academy of Health Sciences, Ghorahi, Dang, Nepal; 2 Karnali Academy of Health Sciences, Jumla, Nepal; 3 Transcultural Psychosocial Organization, Baluwatar, Kathmandu, Nepal

**Keywords:** *anxiety*, *COVID-19*, *mental health*, *pandemic*

## Abstract

**Introduction:**

COVID-19 pandemic and subsequent measures taken by the government to control the situation have imposed adverse impacts on the mental health and wellbeing of the general population. We conducted a study to determine the prevalence of anxiety among general population of a Himalayan district during the COVID-19 pandemic.

**Methods:**

A descriptive cross-sectional study was conducted in rural communities of the Himalayan district during the early stage of the pandemic using the Hospital Anxiety and Depression Scale. Data was collected for a period of one month from 13^th^ April 2020 to 13^th^ May 2020. Ethical approval was sought from the Institutional Review Committee of Karnali Academy of Health Sciences (Reference number: 2076/2077/07). All the participants of age 18 or above were included in the study excluding those with known mental illness. Convenience sampling method was used. A total of 427 participants were included in the study. Data were analyzed using the Statistical Package for the Social Sciences version 20. Point estimate at 95% confidence interval was calculated along with frequency and proportion for binary data.

**Results:**

The prevalence rate of anxiety was 196 (45.9%) (41.15-50.64 at 95% Confidence Interval). Anxiety was more prevalent among farmers, illiterate, widowed, and old aged.

**Conclusions:**

The study indicates a higher prevalence of anxiety amidst the COVID-19 pandemic compared to reported studies and highlights the need for a strategic intervention to promote awareness and wellbeing at mental health level.

## INTRODUCTION

It has been apparent that infectious disease outbreaks like the COVID-19 pandemic can increase morbidity and mortality influencing the global death rate.^1^ If we look back to the previous Severe Acute Respiratory Syndrome (SARS) 2003 or Ebola spread, anxiety, depression, stigma, difficult compliance with quarantine, and development of psychological disorder during recovery have been reported.^2-6^

The post-COVID-19 epidemic in China also has demonstrated more anxiety, depression, and stress.^7,8^ Non-availability of effective treatment along with fear and uncertainty have a significant emotional impact.^9,10^ Additionally, news of fearsome data and difficult control situations along with changed life conditions due to restrictions and lockdown also triggers a crisis situation.^11^ In such circumstances psychological wellbeing could serve as a marker but prevalence estimates are scarce in Nepal and if they are then it is far from the Himalayan people.^12,13^

Hence, this study aimed to find the anxiety level of the general population of Himalayan district of Nepal during the COVID-19 pandemic.

## METHODS

A descriptive cross-sectional study was conducted at the household level in the community of the rural Himalayan district of Nepal. Data collection was done between 13^th^ April 2020 to 13^th^ May 2020. Ethical approval was taken from the Institutional Review Committee of Karnali Academy of Health Sciences (Reference number: 2076/2077/07) prior to the study. Written and verbal consent was taken from each participant.

Author and co-others skilled in mental health research independently collected the data visiting the household at the community level with proper precautions followed as per the government and district administration advice. Participants of age 18 or above and willing to participate in the study were included in the study. Participants with a past history of mental illness were excluded from the study.

The sample size was estimated using proportion by the following formula:

n = Z^2^ × p × q / e^2^

  = (1.96)^2^ × 0.5 × (1-0.5) / (0.05)^2^

  = 385

Where,

n = minimum required sample size
Z = 1.96 at 95% Confidence Interval (CI)
p = prevalence taken as 50% for maximum sample size
q = 1-p
e = margin of error, 5%

Adding 10% non response rate, sample size was calculated to be 427 which were included in the study. Four out of eight (one municipality and seven rural municipalities) were randomly selected using the lottery method. Convenience sampling was done from each municipality and the participants were taken proportinately from them.

A validated Nepali translation version of the Hospital Anxiety Depression Scale (HADS) was used for the estimation of the prevalence of anxiety and depression among the general population.^13^ HADS consists of 14 questionnaires of which seven questions are directed for the estimation of anxiety symptoms and the other seven for the estimation of depressive symptoms on a Likert scale ranging from 0 to 3. In response to questions, participants provide the subjective experience they have been experiencing in the preceding week. The score in each question was zero for none, three for marked symptoms and the total maximum of 21 for each mental health disorder. Of the maximum 21 scores for anxiety symptoms, 0-7 was regarded as normal, 8-10 was regarded as borderline abnormal and 11-21 was regarded as abnormal.

Data entry and analysis were done by using the Statistical Package for the Social Sciences version 20. Point estimate at 95% Confidence Interval was calculated along with frequency and proportion for binary data.

## RESULTS

Among 427 participants, nearly half of the respondents 196 (45.9%) showed abnormal anxiety levels (41.1550.64 at 95% Confidence Interval) (Table 1).

**Table 1:** Prevalence of Anxiety (n = 427).

Description	Categories	n (%)
	Normal	95 (22.24)
Anxiety level	Borderline abnormal	136 (31.85)
	Abnormal	196 (45.90)

In the study male and female participation was 237 (55.50%) and 190 (44.49%) respectively. The mean age was 39.45±16.2 years (18-82). It shows almost half 209 (48.94%) of the participants were from aged 30 to 59 years and more than two-thirds 305 (71.42%) of the participants were married. The majority of the respondents were Chhetri and Brahmin. Hindus were predominant in the locality 405 (94.85%). Farming was the main source of income in the locality with 287 (67.21%). To demonstrate the education level of participants, persons who have never attended school or could not read or write were regarded as illiterate while the rest were categorized within the literate group. The participation from the literate group was higher than 305 (71.42%). Most of the participants 377 (88.29%) had a monthly income of less than 25,000 Nepalese Rupees (Table 2).

**Table 2:** Socio-demographic characteristics (n= 427).

Demographic variables	Categories	n (%)
Gender	Female	190 (44.49)
	Male	237 (55.50)
	Below 30	153 (35.83)
Age	31-59	209 (48.94)
	60 and above	65 (15.22)
	Unmarried	93 (21.8)
Marital status	Married	305 (71.4)
	Widow/Divorced	29 (6.8)
	Brahmin	82 (19.2)
	Chhetri	293 (68.6)
Ethnicity	Janajati	11 (2.57)
	Dalit	34 (7.96)
	Others	7 (1.63)
Religion	Hindu	405 (94.84)
	Others	22 (5.15)
	Farmer	287 (67.21)
Occupation	Government	37 (8.66)
	Non-government	20 (4.68)
	Others	83 (19.43)
Literacy status	Illiterate	122 (28.57)
	Literate	305 (71.42)
	< 25000	377 (88.29)
Income (Rs)	25000-50000	38 (8.89)
	50000-100000	12 (2.81)

Since farmers were the maximum participants in our study a significantly higher level of anxiety marked by abnormal anxiety level in 157 (54.76%) was observed. Those participants who were holding a job in the nongovernment section were having a higher percentage of borderline abnormal anxiety level being 13 (65.00%). Participants having total monthly income between Rs 25000 to Rs 50000 had the least abnormal anxiety level compared to less than Rs 25000 and more than 50000. As with increasing age the abnormal anxiety level was found to be increasing with the age group of more than 60 years and above showing abnormal anxiety level in 42 (64.61%). No significant difference in anxiety level was found between married and unmarried, however, the marked abnormal anxiety level was observed in the widowed and divorced participants, a total of 22 (75.86%). Additionally, a significant abnormal anxiety level was found in 70 (57.37%) illiterate participants compared to literate participants 126 (41.31%) (Table 3).

**Table 3:** Anxiety level in different socio-demographics (n= 427).

Demographic	Categories	Normal n (%)	Borderline Abnormal n (%)	Abnormal n (%)
Sex	Female	39 (20.5)	58 (30.5)	93 (48.9)
	Male	56 (23.6)	78 (32.9)	103 (43.5)
Occupation	Farmer	46 (16.0)	84 (29.3)	157 (54.7)
	Government	10 (27.0)	14 (37.8)	13 (35.1)
	Non-government	3 (15.0)	13 (65.0)	4 (20.0)
	Others	36 (43.4)	25 (30.1)	22 (26.5)
	<25000	81 (21.5)	118 (31.3)	178 (47.2)
Income	25000-50000	11 (28.9)	15 (39.5)	12 (31.6)
	50000-100000	3 (25.0)	3 (25.0)	6 (50.0)
	Below 30	52 (34.0)	46 (30.1)	55 (35.9)
Age	30-59	35 (16.7)	75 (35.9)	99 (47.4)
	60 and above	8 (12.3)	53 (23.1)	42 (64.6)
	Unmarried	30 (32.3)	30 (32.3)	33 (35.5)
Marital Status	Married	62 (20.3)	102 (33.4)	141 (46.2)
	Widow/Divorced	3 (10.3)	4 (13.8)	22 (75.9)
Literacy Status	Illiterate	18 (14.8)	34 (27.9)	70 (57.4)
	Literate	77 (25.2)	102 (33.4)	126 (41.3)

## DISCUSSION

The current study estimates the prevalence rate of anxiety to be 45.9%. Similarly, studies conducted in Nepal by online study also during the COVID-19 pandemic have shown the prevalence of anxiety to be 31.0%.^14^ Compared to this study, our study showed the prevalence of anxiety to be markedly higher. Contrast findings are from a similar study conducted in the Kurdistan region, Iraq during the period of the pandemic that has estimated the prevalence of severe anxiety in the lower side compared to our study.^15^ Assessing anxiety within socio-demographic variables showed a high prevalence of anxiety (54.7%) among farmers compared to others. This is in line that a number of studies have shown high rates of anxiety among farmers.^16^ Findings from the studies like farmers from Canadian study, our prevalence of anxiety is greater as they have reported only 33.2% anxiety.^17^ Interesting is also that, repeated findings in regards of gender that anxiety is more prevalent among females contradicts with our findings that even differing to the southern border India.^15,18^

Compared to the study conducted in China including Hubei province and Wuhan where the virus was identified for the first time the warning vulnerability was directed towards the 21-40 years age group, however, our study indicates the concern towards age 60 and above age group.^19^ Similarly, a study in Iranian population also stressed the concern in the early and mid-adulthood and that is in line with our study as our study also showed increasing vulnerability with increasing age however it differs in that much-needed concern in the middle to a low-income country like Nepal is among the geriatric population.^20^ Most of our study participants were having an income of less than 25,000 Nepalese rupees which is below the standard income, however, did not support the findings from Nigeria that socioeconomic status influences psychological symptoms and mental disorders.^21^ Inline to the Japanese study during this pandemic, our study findings support that those who were having stable income with government jobs were less likely to report anxiety compared to other non-government officials as job security became a threat for most of the persons because of economic crisis due to extending lockdowns.^22^

Our study findings highlight that the prevalence of anxiety is high among illiterate, widowed/divorced. Similar findings were presented in China during this pandemic for the divorced population.^23^ However, most of our study participants were widowed compared to divorced (n= 23 and n= 4). Although the findings discussed herein represent a very small population but can be used to compare and represent similar geographics and socio-economics across the globe. In the context of the remote Himalayas of Nepal, being widowed represents a big socio-cultural challenge, and old age, illiteracy, and farming also overlap in this population. Furthermore, illiterates showing higher prevalence compared to the literate ones also point towards the need for public health measures to promote mental health in this group.

Data is very scarce representing the mental health status of the Himalayan regions within Nepal. Since the study is at the time of pandemic and the nationwide lockdown was already implemented, even for the convenience sampling not exceeding its double fold of the calculated sample size has made the sample size small and one of the limitations of our study. Since there are no previous prevalence studies to compare, our prevalence study during the early dreadful time of pandemic might have been influenced owing to fear and the panic situation created by the pandemic in itself. Hence, much can be learned from larger studies with pre and post-pandemic evaluation to further illustrate the prevalence of anxiety disorder.

## CONCLUSIONS

The prevalence of anxiety among community people of the Himalayan district of Nepal was higher than the estimates from reported studies from other nations as well as international studies.
